# Reconfiguration of Localized Ruthenium Surface via Incorporating Single Platinum Atoms for Favorable Hydrogen Oxidation Catalysis

**DOI:** 10.1002/smll.75024

**Published:** 2026-08-04

**Authors:** Daeil Choi, Dong Wook Lee, Hochang Song, Seung‐hoon Kim, Dohoon Kim, Jongkyung Ryu, Jue‐Hyuk Jang, Sungyong Cho, Sungchul Lee, Segeun Jang, Hyung Chul Ham, Sung Jong Yoo

**Affiliations:** ^1^ Center For Hydrogen and Fuel Cells Korea Institute of Science and Technology Seoul Republic of Korea; ^2^ Energy Policy Research Team Korea Institute of Energy Research Daejeon Republic of Korea; ^3^ Advanced Fuel Cell Technology Development Team 2 Hyundai Motor Group Uiwang Republic of Korea; ^4^ Department of Chemistry and Chemical Engineering Education and Research Center for Smart Energy and Materials Inha University Incheon Republic of Korea; ^5^ School of Mechanical Engineering Kookmin University Seoul Republic of Korea; ^6^ Department of Materials Science and Technology Pohang University of Science and Technology Pohang Republic of Korea; ^7^ Division of Energy & Environment Technology KIST School University of Science and Technology Daejeon Republic of Korea

**Keywords:** CO tolerance, hydrogen oxidation reaction, oxophilicity, single atom alloy, ultralow Pt

## Abstract

Despite the fact that only 2% of active sites can satisfy hydrogen oxidation reaction (HOR) activity thanks to fast kinetics, the fuel cell anode is still dependent on catalysts with large amounts of platinum (Pt). Herein, a minimal‐cost ruthenium catalyst bearing ultralow quantities of Pt single atoms (RuPt_SA_) is developed to provide a high catalytic activity and tolerance to impurities as well as breakthrough reduction in Pt loading amounts. By introducing 1 wt.% Pt as a galvanic replacement for the Ru lattice, the active sites for the adsorption/desorption of hydrogen and CO are redefined. Ru acts both as an electron donor to Pt and as a host for OH groups, thereby accelerating the catalytic process. Using 1 wt.% Pt atoms, the HOR activity and CO resistance of Ru/C are improved, and the HOR mass activity of RuPt_SA_/C is 25.4‐fold higher than that of Pt/C. Synergy between Ru and Pt is demonstrated by density functional theory calculations and verified using practical single‐cell evaluations. Furthermore, RuPt_SA_/C exhibits an 18.4‐fold higher mass activity than Pt/C in the HOR of an anion exchange membrane fuel cell, indicating its promise for use as a universal fuel cell anode catalyst.

## Graphical abstract

The introduction of a Pt single atom changes the Ru surface structure and results in the reconstruction of the active sites for hydrogen and CO oxidation catalysis. With a Pt loading of only 1 wt.%, RuPt_SA_ exhibited superior HOR activity and resistance to impurities compared to commercial Pt catalysts in both acid and alkaline electrolytes.

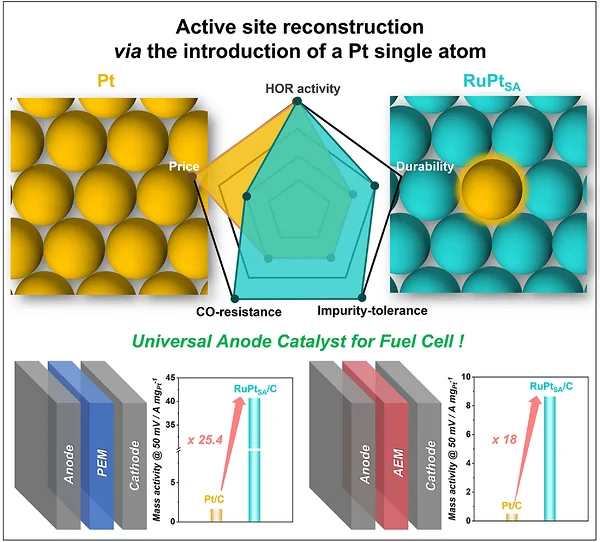

## 1 Introduction

Fossil fuels continue to dominate the global energy system, constituting 81% of the total primary energy demand [1, 2] and causing potentially catastrophic effects on the climate. As such, the transition from a carbon economy to a renewable energy economy has become inevitable. As the world grapples with the urgent need to address climate change and reduce carbon emissions, the hydrogen energy has emerged as a beacon of hope and a pivotal player in the transition toward a carbon‐neutral world. Indeed, by 2050, hydrogen energy has the potential to satisfy 14% of the energy requirements in the United States and 24% of the global energy demand, with an estimated 30% allocated to transportation [3, 4, 5]. Fuel cells are representative energy conversion devices aimed at the consumption of hydrogen as a pollutant‐free energy source for power generation, buildings, and transportation [6, 7, 8]. The transportation sector offers several advantages over conventional gasoline‐powered vehicles, including a higher efficiency, a longer range, rapid refueling, and zero emissions (with the exception of water) [3, 9, 10]. However, despite the potential benefits of fuel cells, there are several technical and economic challenges, aside from the lack of infrastructure, that must be addressed before they can become mainstream energy carriers [11, 12, 13, 14, 15, 16, 17].

Approximately 48% of the hydrogen produced globally originates from reformed hydrocarbons, which contain impurities such as CO_2_, CO, and sulfur, thereby rendering it difficult to directly feed this source into fuel cells. Indeed, fuel cells require hydrogen of an extremely high purity (i.e., >99.97% to be employed) [18]. According to the International Organization for Standardization, the allowable CO concentration in fuel cell hydrogen is 0.2 ppm, above which it is considered a lethal impurity [19]. CO is a strong competitor of hydrogen in hydrogen oxidation catalysis, adsorbing readily onto the surface of the Pt catalyst, which is a common catalyst in proton exchange membrane fuel cells (PEMFCs) and anion exchange membrane fuel cells (AEMFCs) [20, 21]. In fact, even 1 ppm of CO is known to block ∼90% of the Pt active sites [22]. Compared to PEMFCs, in the case of AEMFCs, its HOR kinetics is intrinsically more sluggish in several orders, therefore the catalyst is more susceptible to CO poisoning, and additionally may be exposed to poisoning of organic cations such as tetraalkylammonium [N(CH_3_)_4_^+^] (TMA^+^) originating from membranes or ionomers [23]. As a result of poisoning the fuel cell anode, the fuel cells suffer from a significant performance degradation. To address such issues, RuPt‐ and Pt‐based alloy catalysts are commonly employed since they are less easily contaminated [24, 25]. More specifically, to mitigate CO poisoning, the more oxophilic Ru is considered a desirable counterpart to Pt because it not only tunes the electronic structure of Pt (i.e., via the alloying effect), but it also accelerates the oxidation of CO by adsorbing OH at lower potentials (i.e., via the Langmuir–Hinshelwood mechanism) [26, 27].

Unlike the corresponding oxygen reduction reaction (ORR), the hydrogen oxidation reaction (HOR) is kinetically very fast in PEMFCs, so the presence of only 2% of the active sites can provide adequate HOR activity [28], but a large amount of Pt loading is still required in typical alloy structures. Therefore, single atom‐based catalysts, which can dramatically reduce the amount of Pt used, may be a desirable alternative for anode catalyst preparation. Importantly, these systems have been theoretically and experimentally demonstrated to exhibit activities comparable to those of nano‐sized catalysts. However, because of the high surface free energies associated with single atoms, they tend to readily undergo aggregation during the reaction. Thus, methods to form stable configurations of isolated atoms have been studied and single‐atom alloys (SAAs) have been devised for anchoring to various host metal/metal oxide substrates [29, 30]. For example, the first explicit report of the SAA concept was made by Sykes et al., who prepared Pd/Cu (111) surface alloy materials in which the Pd atoms were dispersed on the Cu (111) plane [31]. SAAs combine the advantages of SACs and alloy materials to present a novel paradigm for catalysis. In addition, SAAs can maximize atomic utilization efficiency of precious metals while keeping their use to a minimum. In such systems, the interactions between the single metal atoms and the host metal at the SAA catalyst interface facilitates a strong synergy, ultimately leading to an excellent catalytic activity and selectivity.

In line with this concept, recent studies have reported various strategies to enhance HOR activity and CO tolerance by introducing isolated Pt atoms into Ru‐based catalysts or by engineering the surface structure, including Ru–Pt SAAs, nanoparticle–single atom hybrid structures, and metal–oxide heterointerfaces [32, 33, 34, 35]. These approaches demonstrate that the interaction between Ru and Pt can regulate hydrogen adsorption/desorption behavior and modify the electronic structure of the catalyst surface [32, 33], thereby improving catalytic performance. In addition, particularly in alkaline environments, the adsorption of OH species is recognized as a key factor governing HOR kinetics, where the competitive adsorption and interaction between hydrogen and OH species play a critical role in determining catalytic activity [34, 35]. However, most of the previous studies have been limited to specific electrolyte conditions or half‐cell evaluations, and validation under practical fuel cell operating conditions remains limited. Therefore, the development of catalysts that can operate efficiently in both acidic and alkaline environments, while maintaining stable performance under practical single‐cell operating conditions, is required.

Herein, we propose a novel SAA RuPt catalyst containing isolated Pt single atoms on Ru nanoparticles (RuPt_SA_). The effects of the ultra‐low Pt content on the HOR performance and the alleviation of CO poisoning are examined, and the synergistic effect between the Pt atoms and the Ru nanoparticles is demonstrated in single‐cell evaluations and density functional theory (DFT) calculations. Subsequently, the prepared RuPt catalyst is applied in a PEMFC and an AEMFC to determine its HOR activity and tolerance to impurities. Overall, we aim to demonstrate the potential of RuPt_SA_ to act as a universal fuel cell anode catalyst.

## 2 Results and Discussion

### 2.1 Preparation and Characterization of the RuPt_SA_/C System

**FIGURE 1:**
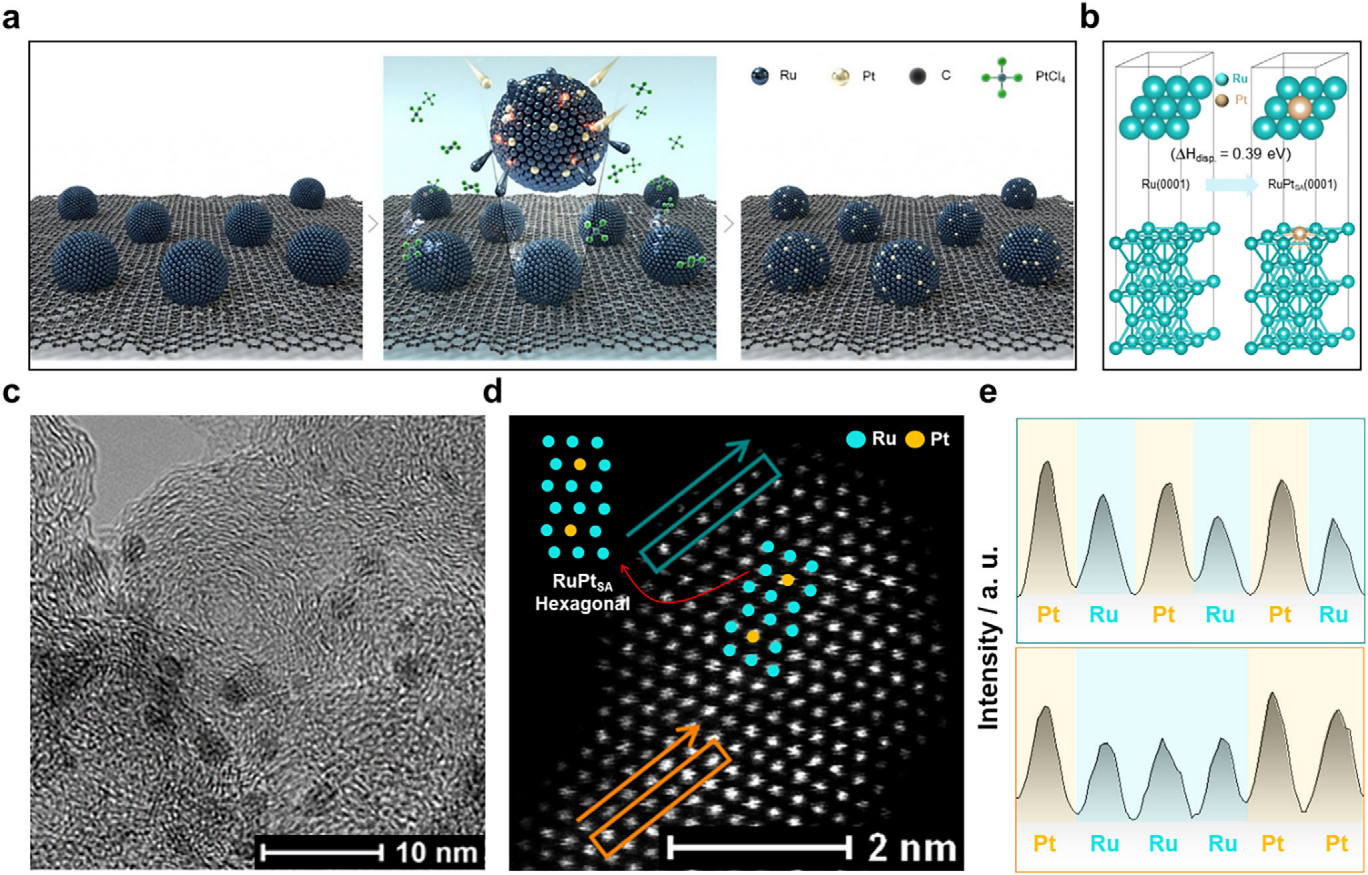
(a) Schematic illustration of synthetic process used to prepare RuPt_SA_/C, which was based on aqueous galvanic replacement. (b) Schematic illustration showing the DFT calculation models indicating that the formation of RuPt_SA_/C by the galvanic replacement of Pt with Ru is a thermodynamically favorable process. (c) TEM image of the prepared RuPt_SA_/C. (d) HAADF‐STEM images of RuPt_SA_ (The green and orange squares indicate the line profiling area). (e) Confirmation of the locational correlation of the Pt single atoms on the Ru surfaces according to the line profiling method.

The RuPt_SA_/C system was prepared using a three‐step synthetic method [36], involving synthesis of Ru/C, heat treatment, and the galvanic replacement of Ru with Pt (see Figure 1a). The spherical Ru/C was synthesized using NaBH_4_ (Figure S1a). Subsequent annealing at 250°C for 1 h under air removed any residual surfactant, while further annealing at 300°C for 1 h under a H_2_ atmosphere promoted reduction of the Ru metal (Figure S1b). Galvanic replacement was then performed to introduce the single Pt atoms onto the Ru/C surface. Due to the different standard reduction potentials of the two metals, some of the Ru species were oxidized and dissolved, and a galvanic replacement reaction occurred to reduce Pt ions to single Pt atoms and fill the voids left by the Ru. The galvanic replacement reaction between Pt and Ru can be described as follows [37, 38]:

$${\left[\mathrm{PtC}l_{4}{\left(\mathrm{OH}\right)}_{2}\right]}^{2-}+4\mathrm{Ru}+2H_{2}O\;\to \;\mathrm{Pt}+4\mathrm{RuOH}+4Cl^{-}+2H^{+}$$

Based on model simulations, the formation of RuPt_SA_/C by the galvanic replacement of Ru with Pt is a thermodynamically favorable process (∆H_disp._ = 0.39 eV), as shown in Figure 1b. Indeed, this process resulted in the introduction of 0.98 wt.% Pt to Ru/C, as confirmed by quantitative analysis (see Table S1). In addition, Figure 1c and Figure S2 show the bright‐field transmission electron microscopy (TEM) images and the particle diameter distribution, respectively, wherein it can be seen that the RuPt_SA_/C particles with an average particle size of ∼3 nm were evenly distributed over the carbon support. Scanning transmission electron microscopy (STEM) images of the prepared RuPt_SA_/C are shown in Figure S3 and Figure 1d, which allow observation of the brighter atomic features attributable to Pt species distributed over the surfaces of the Ru nanoparticles. As can be seen in Figure 1e, the atomic arrangement of RuPt_SA_ corresponded to hexagonal close‐packed Ru (hcp‐Ru), indicating that Pt was successfully anchored to the Ru lattice. To further characterize the atomic introduction of Pt, a line profile (green and orange squares in Figure 1d) was obtained, and the contrast intensities of Pt and Ru were clearly identified, as shown in Figure 1e.

**FIGURE 2:**
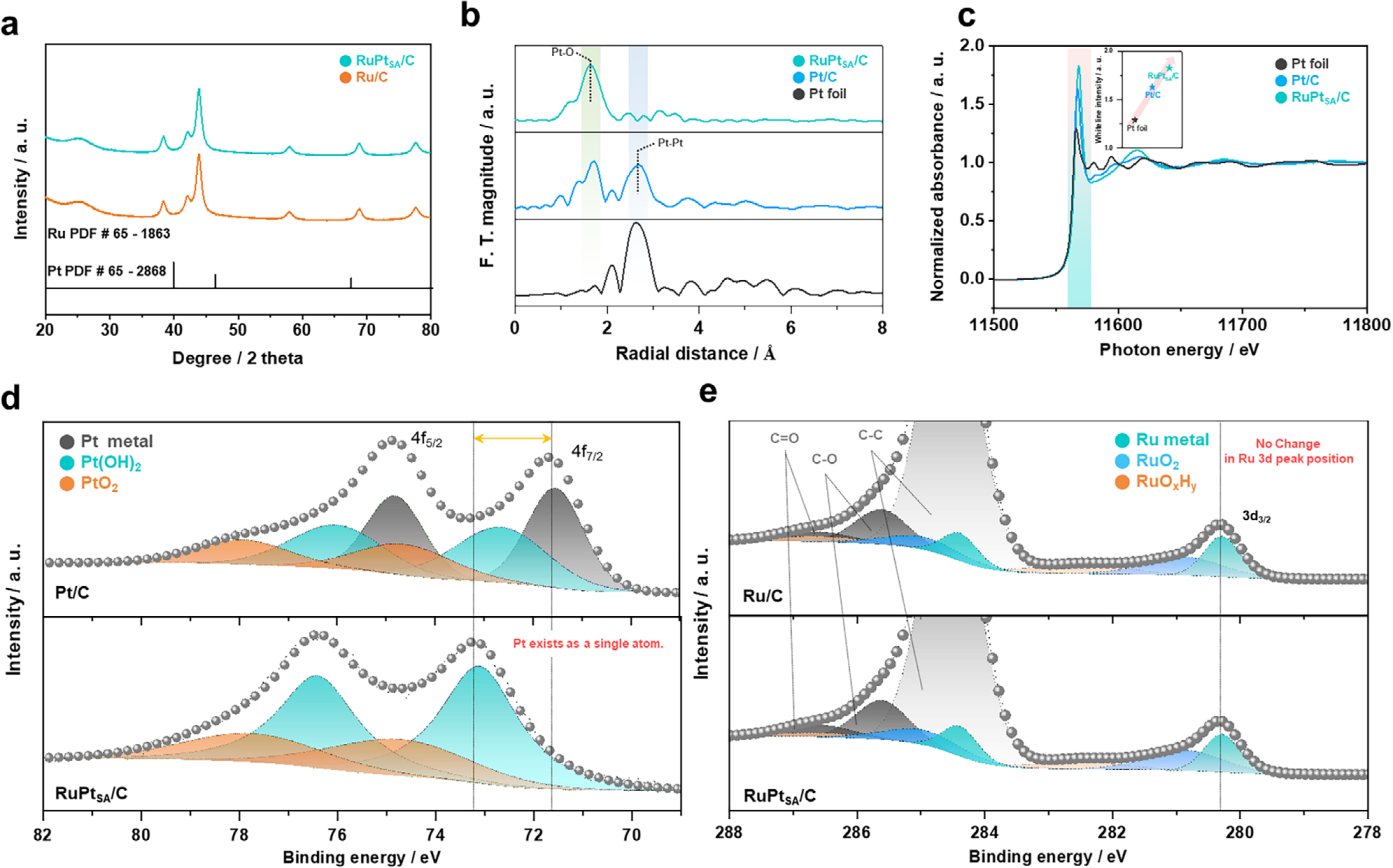
(a) XRD patterns of Ru/C and RuPt_SA_/C with indexing according to the JCPDS cards of Pt and Ru. (b) The k^3^‐weight Fourier transformed EXAFS spectra of a Pt foil, Pt/C, and RuPt_SA_/C. (c) Normalized XANES spectra at the Pt‐L_3_ edge for the Pt foil, Pt/C, and RuPt_SA_/C species. Inset: Comparison of the white‐line intensities of the Pt foil, Pt/C, and RuPt_SA_/C. (d) Pt 4f XPS spectra of Pt/C and RuPt_SA_/C showing deconvolution results for the Pt metal (gray), Pt(OH)_2_ (sky blue), and PtO_2_ (orange) states. (e) Ru 3d XPS spectra for Ru/C and RuPt_SA_/C showing deconvolution results for the Ru metal (sky blue), RuO_2_ (blue), and RuO_x_H_y_ (orange) states.

The crystal structures of Ru/C and RuPt_SA_/C were also analyzed by X‐ray diffraction (XRD, Figure 2a) giving patterns corresponding to hcp‐Ru (PDF No. 65‐163), and indicating that the introduction of Pt had little effect on the Ru crystal structure [39]. These observations indicate that Pt existed in the form of single atoms. Figure 2b shows the Pt L_3_‐edge extended X‐ray adsorption fine structure (EXAFS) results for the prepared RuPt_SA_/C, along with those of the conventional Pt/C and Pt foil. The spectrum recorded for RuPt_SA_/C shows the absence of an oscillation in the high k region (k > 8 Å), indicating the dominance of low Z backscatters, i.e., an oxygen adsorbate in the current case [40]. Correspondingly, in the Fourier‐transformed EXAFS spectrum of RuPt_SA_/C, no peak corresponding to Pt–Pt atomic coordination at ∼2.7 Å was observed (c.f., the spectra of the Pt foil and Pt/C), and instead, a dominant peak was detected at ∼1.7 Å, originating from the Pt–O contribution. These observations clearly indicate that Pt exists in the form of single atoms in the RuPt_SA_/C catalyst structure. X‐ray absorption near‐edge spectroscopy (XANES) and X‐ray photoelectron spectroscopy (XPS) were performed to further elucidate the single‐atom nature of Pt in the RuPt_SA_/C system, and to closely investigate the electronic structure of the catalysts. It is generally known that the white‐line intensity of a XANES spectrum is closely related to the oxidation state of the corresponding species. Thus, as shown in Figure 2c, which displays the Pt L_3_‐edge XANES spectra of RuPt_SA_/C, Pt/C, and a Pt foil, the white‐line intensity of RuPt_SA_/C is significantly higher than those of the Pt/C and Pt foil species (see Figure 2c, inset), thereby suggesting that the Pt species in RuPt_SA_/C exist in a more oxidized state [41]. These characteristics were also observed in the XPS. More specifically, Figure 2d shows the deconvoluted Pt 4f XPS spectra of Pt/C and RuPt_SA_/respectively, wherein it can be seen that the doublet associated with RuPt_SA_/C was present at a higher binding energy. The deconvolution results were displayed in Figure S4 and Table S2: Only areas corresponding to Pt(OH)_2_ and PtO_2_ were observed, and no peak corresponding to metallic Pt was present. These XPS results are consistent with the EXAFS and XANES results, thereby indicating that the Pt species in RuPt_SA_/C are present as oxidized forms (e.g., Pt^2+^ and Pt^4+^), and further confirming that Pt exists as in the single‐atom state [41]. Furthermore, Ru 3d XPS analysis was performed to identify changes in the electronic structure of Ru, and it was found that the peak binding energies of Ru/C and RuPt_SA_/C were practically identical (see Figure 2e). The deconvolution results show that the content of metallic Ru was slightly reduced in RuPt_SA_/C after galvanic replacement (see Figure S4 and Table S3). It was therefore considered that electrons from the Ru host are donated to Pt, and that the metallic Ru partially transitions into an oxidized state. The lack of any significant change in the binding energy of the Ru 3d species and the localized change observed in the Ru component fraction can be attributed to the formation of localized SAAs in the Ru host containing an ultralow amount of Pt.

### 2.2 Hydrogen Oxidation and CO Tolerance of the RuPt_SA_/C Catalyst

**FIGURE 3:**
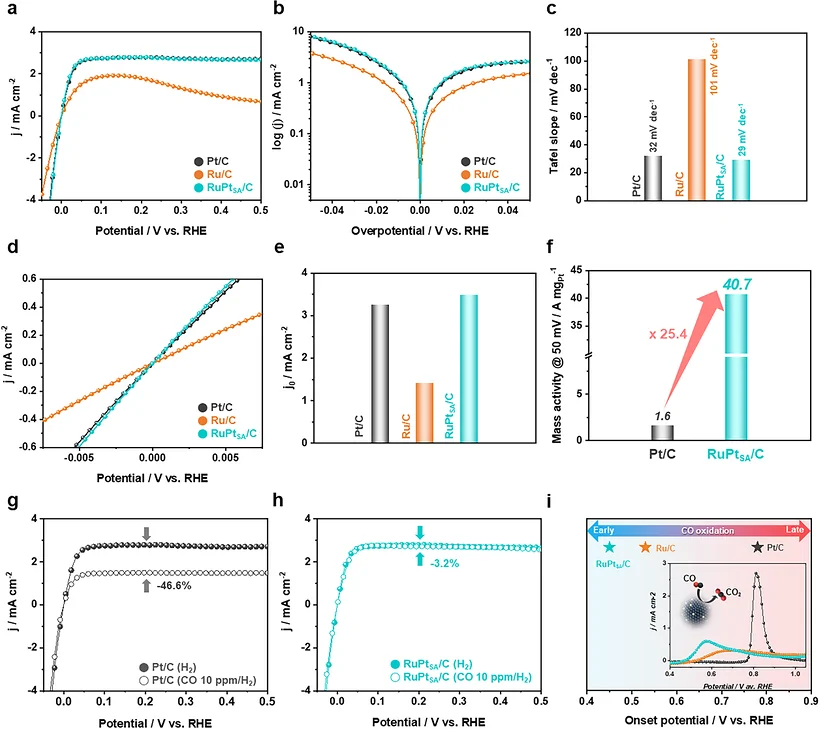
(a) HOR polarization curves recorded for the Pt/C, Ru/C, and RuPt_SA_/C catalysts. All measurements were conducted in H_2_‐saturated 0.1 M HClO_4_ with a rotation speed 1600 rpm at 4°C. (b) Tafel plots and (c) slopes recorded for Pt/C, Ru/C, and RuPt_SA_/C. (d) Micro‐polarization regions of the HOR polarization curves for the Pt/C, Ru/C, and RuPt_SA_/C species. (e) Exchange current densities of the Pt/C, Ru/C, and RuPt_SA_/C catalysts calculated using the simplified Butler–Volmer equation in the micro‐polarization region. (f) Pt mass‐normalized activities of Pt/C and RuPt_SA_/C at 50 mV. (g, h) HOR polarization curves of Pt/C and RuPt_SA_/C in H_2_ and CO 10 ppm/H_2_ –saturated 0.1 M HClO_4,_ respectively. (i) Onset potentials for the oxidation of CO over the Pt/C, Ru/C, and RuPt_SA_/C catalysts. Inset: Background‐corrected CO stripping results measured at a scan rate of 20 mV s^−1^ after monolayer CO adsorption.

The HOR polarization curve of RuPt_SA_/C is presented in Figure 3a along with those of the Pt/C and Ru/C systems for comparison. It can be seen that the HOR activity of Ru/C decreased rapidly from 0.2 V (vs. the reversible hydrogen electrode, RHE) because the Ru surface was covered with oxygen species due to its strong oxophilicity [42]. However, the introduction of even trace amounts of Pt changed the intrinsic electrochemical properties of the Ru, leading to a dramatic increase in the HOR activity. This improvement can be attributed to the introduction of Pt atoms, which can promote active site switching, i.e., where hydrogen preferentially adsorbs and desorbs from Ru to Pt, and Ru provides electrons to Pt. In addition, RuPt_SA_/C exhibited an HOR activity comparable to that of Pt/C despite containing a significantly lower Pt loading than Pt/C. The excellent HOR kinetics of this catalyst were also supported by the Tafel plots and slopes provided in Figure 3b,c. To further investigate the HOR kinetics, the exchange current densities of the samples were compared in the micro‐polarization region, as shown in Figure 3d,e. Since charge transfer is the rate‐determining step in electrochemical reactions owing to the sufficiently fast mass transport rates, the exchange current density (*j_0_*) was derived for each HOR (i.e., for the Pt/C, Ru/C, and RuPt_SA_/C catalysts) using the Butler–Volmer equation, as follows:

(1) $$j_{k}=j_{0}\;\left[e^{-\frac{\alpha F\eta }{RT}}-e^{\frac{\left(1-\alpha \right)F\eta }{RT}}\right]$$

where α is the transfer coefficient, F is Faraday's constant, η is the overpotential, R is the gas constant, and T is the temperature. In particular, for a range of potentials satisfying the Butler–Volmer equation, j is linearly proportional to η in the very low overpotential region close to the HOR equilibrium potential of −5 to 5 mV, as follows [43, 44]:

(2) $$j_{k}=j_{0}\;\frac{F\eta }{RT}$$

This extremely low potential region is referred to as the micro‐polarization region, and the exchange current density can be obtained as the slope of the *j–η* curve in this region. Thus, the calculated values of *j_0_* were 1.41, 3.24, and 3.47 mA cm^−2^ for Ru/C, Pt/C, and RuPt_SA_C, respectively (Figure 3e). In addition, Figure 3f shows a comparison of the HOR kinetic current densities at 50 mV normalized to the amount of Pt. As indicated, the RuPt_SA_/C catalyst (40.7 A mg_Pt_ @ 50 mV) exhibited a significantly higher mass activity (i.e., 25.4×) than the Pt/C catalyst (1.6 A mg_Pt_ @ 50 mV), which implies a significant economic advantage in addition to a higher activity. Interestingly, the synergy between the Ru host and the individual Pt atoms (1 wt.%) led to an HOR activity that should be associated with a >20 wt.% Pt loading.

Under hydrogen‐saturated conditions in the presence of 10 ppm CO, RuPt_SA_/C exhibited superior CO avoidance properties than Pt/C (see Figure 3g,h). The HOR performances of Pt/C and RuPt_SA_/C were comparable in a pure H_2_‐saturated atmosphere; however, completely different hydrogen oxidation behaviors were observed in the presence of 10 ppm CO, even in the kinetic region (Figure S5). More specifically, after exposure to CO, the limiting current density of Pt/C decreased by 46.6%, whereas that of RuPt_SA_/C decreased by 3.2%. It should be noted here that a decrease in the limiting current density indicates a decrease in the access of hydrogen to the surface, which can be attributed to the differences in the degree of CO adsorption on the surface. The above results therefore suggest that a lesser amount of CO was adsorbed on the surface of the RuPt_SA_/C catalyst (c.f., Pt/C), indicating that this catalyst is more resistant to poisoning. Similarly, the current density of RuPt_SA_/C at 50 mV was found to decrease slightly from 2.62 to 2.59 mA cm^−2^, while that of Pt/C decreased from 2.6 to 1.4 mA cm^−2^ (i.e., a 46% reduction) (Figure S6). The CO‐tolerant behavior of RuPt_SA_/C was also explored under CO stripping and bulk oxidation conditions, and the related potentials are presented in Figure 3i and Figure S7 and Table S4. It can be seen from the results presented that the oxidation of CO on the RuPt_SA_/C surface is initiated at ∼0.45 V, which represents a significant acceleration compared that observed for Pt/C (i.e., initiation at 0.78 V). As in the case of the HOR, the introduction of individual Pt atoms led to the active site for CO adsorption and desorption being switched from Ru to Pt. In addition, the more oxophilic Ru appeared to preferentially adsorb oxygen species, promoting earlier CO oxidation on Pt; this is known as the Langmuir–Hinshelwood mechanism [27]. In addition to the oxophilic effect, the conventional Ru host may also modulate the electronic structure of Pt by donating electrons to the 5d orbital of Pt, lowering the intrinsic CO adsorption energy and consequently leading to earlier CO oxidation. The change in the catalytic role due to the introduction of Pt favorably enhanced the electrochemical reactivity of the catalyst, and this trend was also observed for the bulk oxidation of CO (Figure S7 and Table S4). More specifically, Figure S8 shows the time‐transient CO bulk oxidation results for the RuPt_SA_/C and Pt/C catalysts at 0.5, 0.6, 0.7, and 0.8 V, respectively. It can be seen that no CO oxidation current is evident at 0.5, 0.6, and 0.7 V for Pt/C, while a current is observed at 0.8 V. In contrast, a CO oxidation current is observed for the RuPt_SA_/C system at 0.5 V. These results suggest that in the absence of Ru, the oxygen species must be exposed to an applied potential of at least 0.8 V to sufficiently form OH_ad_ on the Pt surface and generate a CO oxidation current. However, as mentioned above, the CO oxidation current in RuPt_SA_/C was generated at a lower potential, and was higher than that observed for Pt/C at all potentials; these results were attributed to the intrinsic characteristics of the Ru host. More specifically, Ru is well‐known for its ability to change the O─H bond strength of the H_2_O molecule, which can induce proton conduction within the surrounding water [45]. In this context, Zei et al. reported that anodic polarization weakens both the Ru─CO and Ru─O bonds, thereby indicating that the adsorption of hydroxyl (OH) species becomes possible even when the Ru surface is covered with CO and when the Ru─O bonds are sufficiently weakened to enable CO_2_ formation [38, 42]. Therefore, even at lower potentials, the rich OH adsorbate present on the Ru surface allows the earlier oxidation of CO by Pt. Furthermore, to evaluate the electrochemical stability during the HOR carried out in the presence of 10 ppm CO, chronoamperometry measurements were performed at 0.1 V vs. RHE for 10 000 s. Considering the responses displayed in Figure S9, it is clear that compared to the initial HOR current, the current of RuPt_SA_/C decreased by 19.5%, whereas that of Pt/C decreased by 42.6%. These electrochemical evaluations therefore demonstrate that RuPt_SA_/C outperforms Pt/C in terms of both its CO resistance and its HOR stability in the presence of CO. Moreover, it is clear that the combination of a Ru host with individual Pt atoms is a promising strategy for developing hydrogen oxidation catalysts to incorporate into fuel cells.

### 2.3 Theoretical Prediction of the Hydrogen Oxidation Capability and the CO Tolerance of the RuPt_SA_/C Catalyst

**FIGURE 4:**
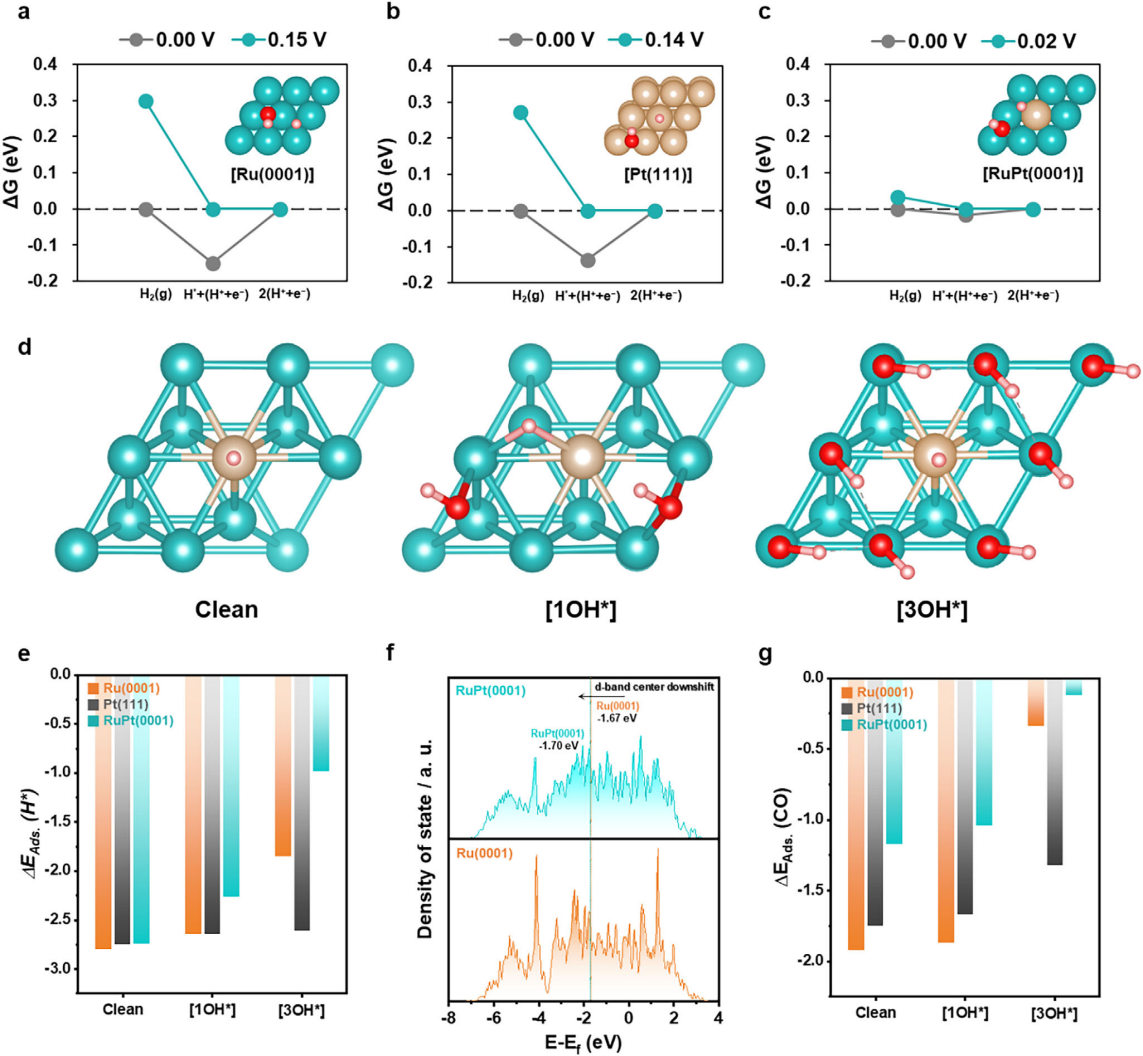
Calculated free energy diagrams for the (a) Ru(0001) slab, (b) Pt(111) slab, and (c) RuPt(0001) slab surfaces. (d) Optimized structure of the H*‐adsorbed RuPt(0001) slab model. (e) H* adsorption energies of Ru(0001), Pt(111), and RuPt(0001) depending on the number of OH groups. (f) Projected d‐band densities of states for the active sites of the RuPt(0001) and Ru(0001) slab models. (g) CO adsorption energies on the Ru(0001), Pt(111), and RuPt(0001) surfaces depending on the number of OH groups.

To elucidate the active species during hydrogen and CO oxidation catalysis in conjunction with the experimental results, three models were designed, namely Ru(0001), Pt(111), and RuPt(0001). Figure 4a–c display the Gibbs free energy changes for the HORs carried out according to these models, wherein it can be seen that in both the Ru(0001) and RuPt(0001) systems, the hydrogen atom was most stably adsorbed at a three‐fold hollow site, whereas on the Pt(111) surface, H was adsorbed on the Pt top site. In addition, it was deduced that RuPt(0001) presented the lowest HOR potential (0.02 V) compared to Ru(0001) (0.15 V) and Pt(111) (0.14 V), suggesting that this potential was strongly associated with the hydrogen adsorption energy of the surface. Thus, RuPt(0001) structures were further designed to adjust the number of adsorbed OH groups, as shown in Figure 4d, and their surface hydrogen adsorption energies were derived and compared with those of the Ru(0001) and Pt(111) models (Figure 4e and Table S5). As indicated, the hydrogen adsorption energies for all surfaces tended to decrease as the number of OH groups increased, and the weakest hydrogen adsorption was observed on the RuPt(0001) surface. The theoretical result that RuPt(0001) exhibits a lower hydrogen adsorption energy (especially in OH‐rich environments) appears to correlate with the excellent HOR activity of RuPt_SA_. Although it is ideal to obtain a moderate hydrogen adsorption energy that is neither too strong nor too weak, a lower adsorption energy generally leads to faster desorption and more rapid kinetics. Interestingly, when single Pt atoms were introduced into the Ru(0001) model, hydrogen was preferentially adsorbed at the Pt sites, suggesting that the adsorption/desorption sites for hydrogen switched from Ru to Pt, and that the catalyst surface was reconstructed. Thus, to identify the electronic structure changes caused by the introduction of Pt, the projected densities of state were compared for the Ru(0001) and RuPt(0001) surfaces, as shown in Figure 4f. It was revealed that the d‐band center of the RuPt(0001) surface (µ = −1.70 eV) was slightly downshifted away from the Fermi level compared to that of Ru(0001) (µ = −1.67 eV) owing to the electron transfer taking place from Ru to Pt. This localized tuning of the electronic structure contributes to the weakening of the hydrogen adsorption energy, resulting in an excellent hydrogen oxidation reactivity. This trend was also confirmed using CO adsorption energy calculations and by comparing the CO affinities of Ru(0001), Pt(111), and RuPt(0001). As shown in Figure S10, a CO molecule adsorbs on the Pt top site on the Pt(111) surface, whereas it adsorbs at the Ru hollow sites on the Ru (0001) and RuPt (0001) surfaces. Under both clean and OH conditions, the RuPt(0001) surface exhibited the lowest CO adsorption energy among the three models (Figure 6g and Table S5), indicating rapid CO desorption from this surface. Indeed, this correlates with the earlier CO oxidation observed for the RuPt_SA_ system (c.f., Ru/C and Pt/C, Figure 3i). In other words, the surface structure of RuPt_SA_ was reconstructed to remove CO from the surface as quickly as possible, endowing it with an excellent CO tolerance. In addition, it was found that the CO adsorption energy can be affected by OH adsorption, wherein OH is preferentially adsorbed on the Ru hollow sites. It should be noted here that the ΔE_Ads._(OH) values for RuPt(0001) on the Pt‐top, RuPt‐bridge, and Ru‐hollow sites are −1.71, −2.12, and −2.75 eV, respectively. It is therefore apparent that the strongly adsorbed OH present at the Ru‐hollow sites can reduce ΔE_Ads._(CO) by interfering with the electronic “back‐bonding” contribution on the RuPt(0001) surface [42].

### 2.4 Single‐Cell Evaluation for Practical Application of the RuPt_SA_/C Catalyst

**FIGURE 5:**
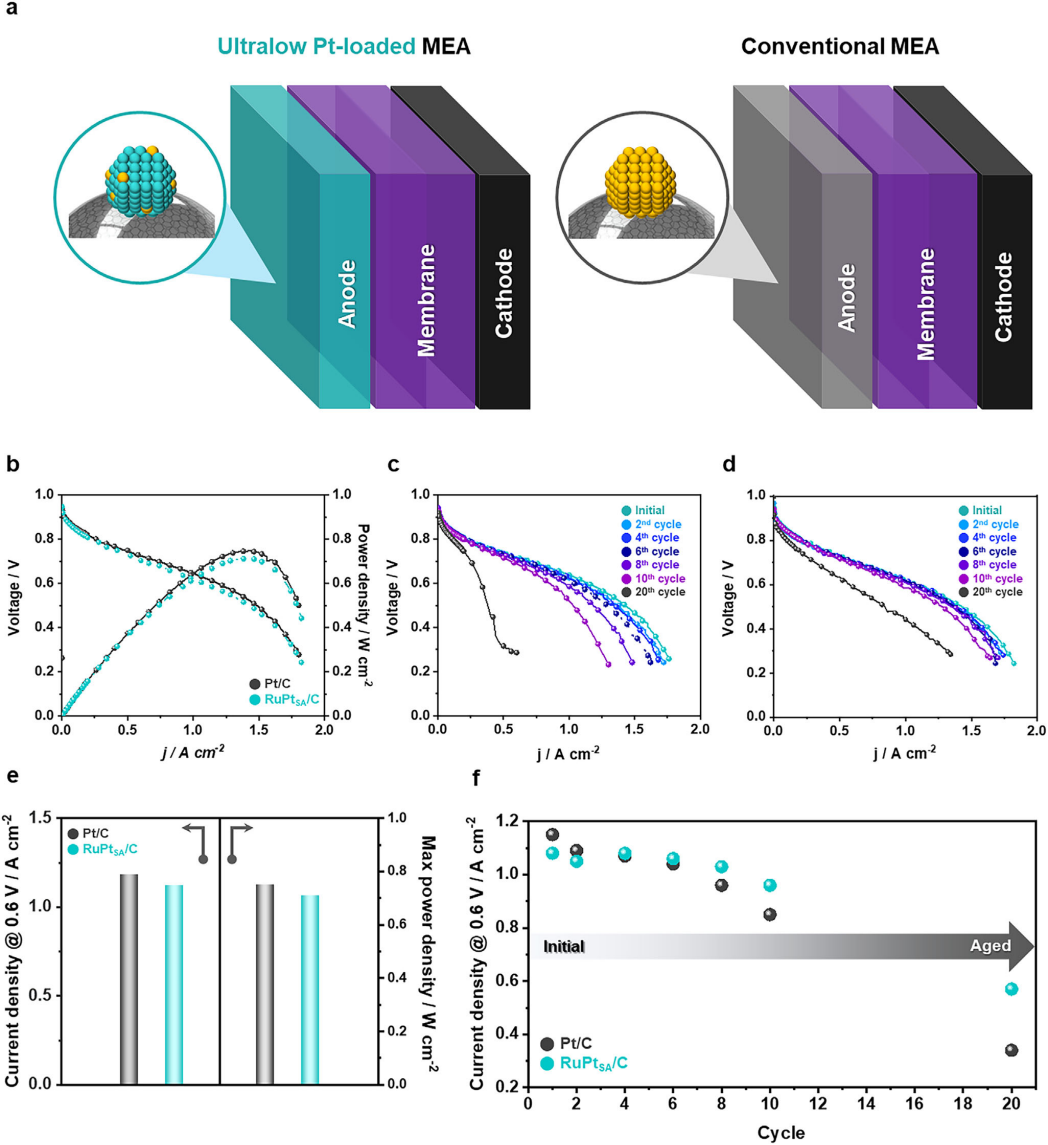
(a) Schematic illustrations of the ultralow Pt‐loaded (RuPt_SA_/C) and conventional (Pt/C) MEAs. (b) *I–V* polarization curves for the Pt/C and RuPt_SA_/C systems under pure H_2_ conditions (operating conditions: 60°C with 100% humidified H_2_ and air flows). (c, d) *I–V* polarization curves for the Pt/C and RuPt_SA_/C systems in a 10 ppm CO/H_2_ environment. (e) Numerical comparison of the current densities at 0.6 V and the maximum power densities under pure H_2_ conditions for the Pt/C and RuPt_SA_/C systems. (f) Change in the current density at 0.6 V for the Pt/C and RuPt_SA_/C systems during cycling in a 10 ppm CO/H_2_ environment.

A single‐cell experiment was conducted to investigate the effect of applying RuPt_SA_/C in a practical fuel cell device. Figure 5a shows a schematic illustration of an ultralow Pt‐loaded membrane electrode assembly (MEA) (RuPt_SA_) and a conventional MEA (Pt/C). The current densities of the ultralow Pt‐loaded MEA and the conventional MEA were determined to be 1.18 and 1.12 A cm^−2^, while their maximum power densities were 0.75 and 0.71 W cm^−2^, respectively. In addition, it was found that the ultralow Pt‐loaded MEA presented a comparable performance to the conventional MEA (Figure 5b,e) under pure H_2_ feed conditions. Interestingly, when 10 ppm CO/H_2_ gas was injected, a distinctly different behavior was observed, as shown in Figure 5c,d. More specifically, the performance of the conventional MEA decreased significantly in the presence of CO, whereas the ultralow Pt‐loaded MEA maintained its initial performance until the 10th cycle. After the 20th cycle, the ultralow Pt‐loaded MEA also exhibited a decreased performance, but continued to outperform the conventional MEA (Figure 5f). These results demonstrate that RuPt_SA_ exhibited a performance comparable to that of Pt/C, but that its resistance to CO poisoning was superior, even in practical evaluations beyond those of the half‐cell.

### 2.5 Hydrogen Oxidation Reaction and Impurity Tolerance of the RuPt_SA_/C Catalyst in an Alkaline Medium

**FIGURE 6:**
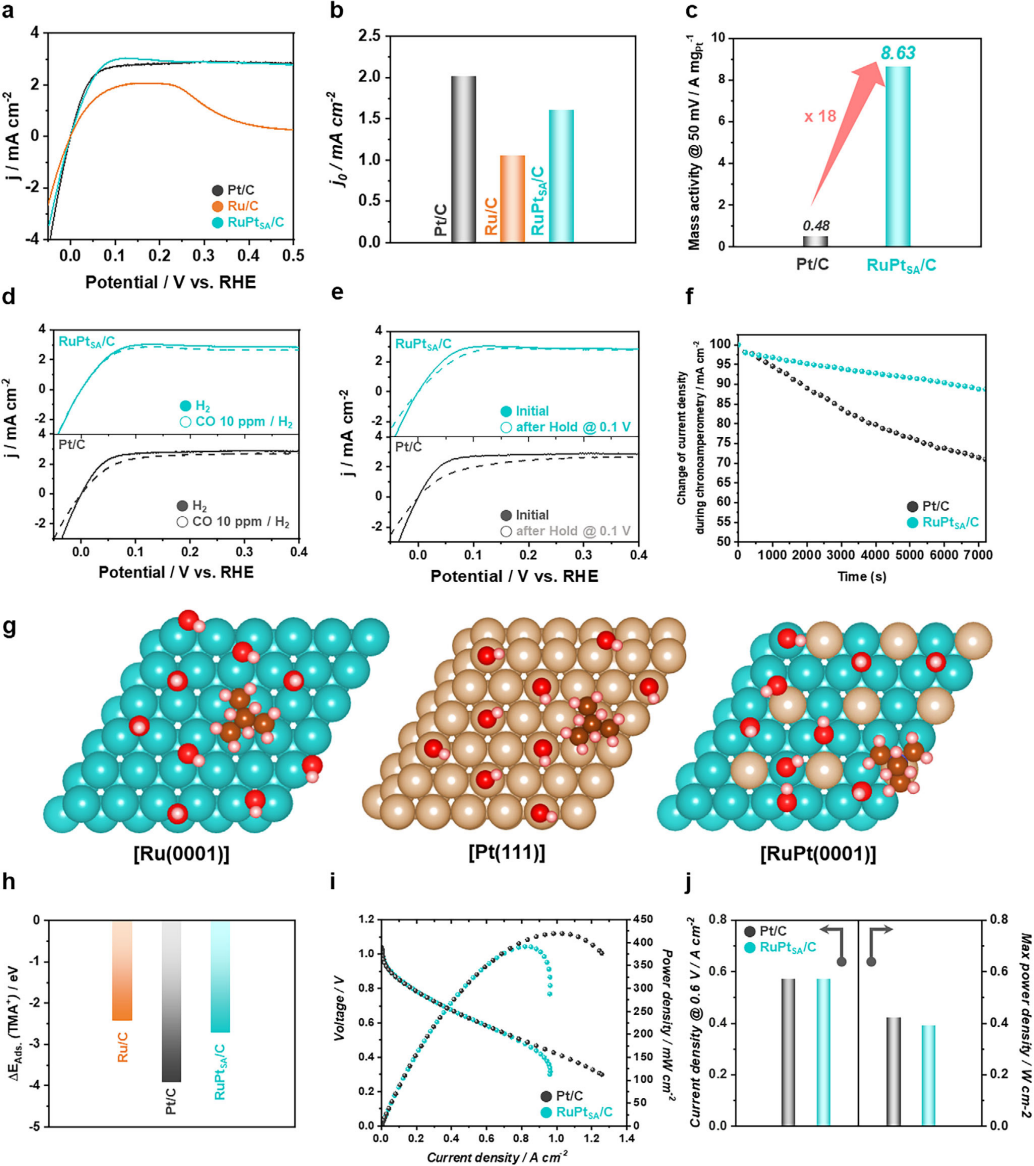
(a) HOR polarization curves for the Pt/C, Ru/C, and RuPt_SA_/C catalysts. All measurements were conducted in H_2_‐saturated 0.1 M KOH with a rotation speed of 1600 rpm at 25°C. (b) Exchange current densities of the Pt/C, Ru/C, and RuPt_SA_/C catalysts calculated using the simplified Butler–Volmer equation in the micro‐polarization region. (c) Pt mass‐normalized activities of Pt/C and RuPt_SA_/C at 50 mV. (d) HOR polarization curves recorded for Pt/C and RuPt_SA_/C in pure H_2_ and in a 10 ppm CO/H_2_–saturated 0.1 M KOH system. (e) HOR polarization curves of Pt/C and RuPt_SA_/C before and after chronoamperometry at 0.1 V for 2 h in H_2_‐saturated 0.1 M TMAOH with a rotation speed of 1600 rpm at 25°C. (f) Variation in the current densities of Pt/C and RuPt_SA_/C during chronoamperometry. (g) DFT calculation models for Pt/C, Ru/C, and RuPt_SA_/C upon the adsorption of quaternary ammonium cations. (h) Adsorption energies of the quaternary ammonium cations on the Ru/C, Pt/C, and RuPt_SA_/C surfaces. (i) *I–V* polarization curves for Pt/C and RuPt_SA_/C under pure H_2_ conditions (operating conditions: 60°C with 100% humidified H_2_ and air flows). (j) Numerical comparison of the current density at 0.6 V vs. the maximum power densities of Pt/C and RuPt_SA_/C.

AEMFCs have gained considerable attention comparable to that of PEMFCs because of the availability of non‐precious metals as cathode catalysts for the oxygen reduction reaction (ORR) [46]. However, in alkaline electrolytes, the HOR kinetics are theoretically over two orders of magnitude more sluggish than those in acidic electrolytes because of the presence of hydroxide ions [47]. Despite the mechanistic disadvantages of the alkaline HOR system, RuPt_SA_/C demonstrated a superior HOR activity and CO tolerance than the Ru/C and Pt/C systems, even in AEMFCs. More specifically, Figure 6a shows the HOR polarization curves for RuPt_SA_/C, Ru/C, and Pt/C. It can be seen that in the presence of ∼1 wt.% Pt atoms, the HOR performance of the catalyst was comparable to that of Pt/C, and represented a remarkable performance improvement over Ru/C. Subsequently, the HOR activity was scrutinized by comparing the quantified HOR kinetics. As a result, the Tafel slope of RuPt_SA_/C was calculated to be 42 mV dec^−1^, which is a dramatic improvement compared to that of Ru/C (i.e., 99 mV dec^−1^, see Figure S11). Similarly, the exchange current densities recorded in the micro‐polarization regions also demonstrate a significant improvement in the kinetics of the RuPt_SA_/C system compared to that of the Ru/C system (Figure 6b and Figure S12). This reveals that the HOR kinetics were intrinsically improved by the introduction of individual Pt atoms. As observed in acidic electrolytes, the electronic interaction between Ru and Pt and the oxophilicity of the Ru nanoparticles promote intrinsic hydrogen oxidation catalysis in alkaline electrolytes. Although there is some debate regarding the participation of OH groups in the HOR reaction mechanism, it is generally accepted that the alkaline HOR activity is governed by the OH binding energy (OHBE), as well as the hydrogen binding energy (HBE) of the catalyst. As previously reported, Ru exhibits OH and hydrogen binding energies at low anode potentials [48]. In the case of RuPt_SA_/C, the intrinsic HBE change of Pt upon electron transfer from Ru to Pt contributes to an enhanced activity; however, in OH‐rich environments, Ru is expected to play a dominant role in the hydrogen oxidation pathway, coupling the adsorbed OH to the hydrogen adsorbed on the single Pt atoms to promote hydrogen oxidation catalysis. This can also be inferred from the computational results (Figure 4e). More specifically, as the degree of OH adsorption increased, the hydrogen adsorption energies of the catalysts decreased; in particular, those of Ru(0001) and RuPt(0001) decreased more significantly than that of Pt(111). This demonstrates that the degree of OH adsorption, especially in Ru‐containing structures, has a significant influence on the hydrogen adsorption energy, which is one of the determinants of the HOR activity. This finding is consistent with previous studies highlighting the critical role of OH species in alkaline HOR kinetics, particularly in regulating the balance between hydrogen‐ and hydroxyl‐related surface intermediates [34, 35]. Even at practical values, RuPt_SA_/C exhibited an 18‐fold Pt mass‐normalized activity compared to that of Pt/C, which was attributed to the loading of single Pt atoms (Figure 6c). To further elucidate the influence of the interactions between the Ru host and the single Pt atoms, the HOR activity of Pt_SA_/C was evaluated (i.e., excluding Ru, see Figure S13). To demonstrate that Pt_SA_/C contains single Pt atoms, the morphology, atomic coordination, and electronic structure of the catalyst were analyzed (Figures S14–S16), and the typical characteristics of single atoms were observed. Interestingly, Pt_SA_/C rarely exhibited activity for the HOR, indicating that Ru plays a crucial role in HOR catalysis by interacting with Pt. Thus, when considered alone, Ru nanoparticles and single Pt atoms exhibit poor HOR activities, while RuPt_SA_/C, in which the two species are combined to generate a harmonic structure, exhibits an excellent catalytic activity.

The complementary behavior of the Ru host and the single Pt atoms is also noteworthy when considering their resistance to impurities. For this evaluation, the HOR polarization curves of Pt/C and RuPt_SA_/C under hydrogen saturation in the presence and absence of CO were recorded, and the results are presented in Figure 6d. In the case of the Pt/C catalyst, the HOR current density decreased noticeably in the presence of CO, whereas the RuPt_SA_/C catalyst exhibited similar HOR activity in both the presence and absence of CO. In the kinetic region, RuPt_SA_/C exhibited a constant HOR slope (*j* vs. potential) despite CO exposure (Figure S17). In a quantitative context, the current density of Pt/C at 50 mV decreased by 23.1% upon exposure to CO (i.e., from 2.29 to 1.76 mA cm^−2^), while that of RuPt_SA_/C decreased by only 4% (i.e., from 2.23 to 2.14 mA cm^−2^ (Figure S18). This result is consistent with theoretical CO adsorption energy calculations. Moreover, as shown in Figure 4g, RuPt(0001) exhibits the lowest CO adsorption energy, regardless of the presence of OH groups, thereby confirming that it exhibits a lower CO affinity in OH‐rich environments, such as alkaline electrolytes. Interestingly, the affinity of Ru toward CO was noticeably altered upon varying the degree of OH adsorption on the surface. Comparing the CO adsorption energies with respect to the adsorption of 1 and 3 OH groups per unit, the CO adsorption energies of Ru(0001) and RuPt(0001) were significantly lower. This means that the intrinsic CO adsorption/desorption energy of Ru is particularly affected by OH adsorption because of its more oxophilic nature, which promote the vigorous participation of the OH group in the CO oxidation reaction. When considering the enhanced CO poisoning tolerance of RuPt_SA_/C under alkaline conditions, the adsorption of OH by Ru is expected to have played a crucial role in the earlier oxidation of CO, although the influence of the localized surface reconstruction (e.g., changes in the electronic structure or active site) must also be considered, as under acidic conditions. In AEMFCs, bulky organic cations that may be contained in the binder can also be adsorbed onto the catalyst surface and inhibit hydrogen oxidation. Thus, to examine how the HOR performance is influenced by cation adsorption, the HOR activities of the catalysts were assessed using a 0.1 M tetramethylammonium hydroxide (TMAOH) electrolyte containing quaternary ammonium cations. Figure 6e shows the polarization curves recorded for the HORs of the Pt/C and RuPt_SA_/C catalysts in H_2_‐saturated 0.1 M TMAOH, both before and after performing chronoamperometry at 0.1 V vs. RHE for a duration of 2 h. The changes in the current densities following chronoamperometry are displayed in Figure 6f, wherein it can be seen that Pt/C and RuPt_SA_/C exhibited reductions of 29.9 and 11.3%, respectively, compared to their initial values. In the presence of TMA cations, the HOR activity of Pt/C was inhibited to a greater extent than that of the RuPt_SA_/C catalyst. Since the intrinsic properties of the catalyst surface were expected to influence the cation adsorption energy, the adsorption energies of the TMA cations [i.e., ΔE_ads_(TMA^+^)] were computed on the Ru(0001), Pt(111), and RuPt(0001) surfaces. Following careful examination of Figure 6g, the three catalyst models were expanded using a (6 × 6) supercell, which is sufficient to adsorb TMA^+^ without any interference from periodic cells. Subsequently, each surface was covered with randomly placed hydroxyl (OH*) groups at 25% coverage. Consequently, it was confirmed that the Pt top site functions as an active site for TMA^+^ adsorption on the RuPt(0001) (RuPt_SA_) surface, leading to a moderate TMA^+^ affinity [ΔE_ads_(TMA^+^) = −2.73 eV], which falls between those of Pt(111) [ΔE_ads_(TMA^+^) = −3.92 eV] and Ru(0001) [ΔE_ads_(TMA^+^) = −2.43 eV] as shown in Figure 6h. This result implies that the isolated Pt atoms on the Ru nanoparticle surfaces renders the catalyst surface more resistant to bulky cations than when Pt metal is present; this is consistent with our experimental data. Notably, the TMA cations were adsorbed significantly more strongly on Pt(111) than on the Ru models (i.e., Ru(0001) and RuPt(0001)). It is therefore evident that Pt‐based catalysts are more susceptible to cation adsorption than Ru‐based catalysts. It must also be considered that the introduction of Pt atoms leads to local reconstruction of the Ru(0001) surface, resulting in a slightly higher TMA^+^ adsorption energy for the RuPt(0001) surface; however, RuPt(0001) exhibits significantly more favorable hydrogen and CO adsorption energies than Ru(0001). Thus, the corresponding RuPt_SA_/C catalyst exploits the intrinsic properties of both Pt and Ru, resulting in a balanced HOR activity and impurity resistance, and indicating its potential for use as a fuel cell anode catalyst, even in the presence of ultralow Pt loadings.

To further evaluate the potential of RuPt_SA_/C (ultralow Pt‐loaded MEA) for use in practical AEMFCs (see Figure 5a), a single‐cell evaluation was performed and compared with that of a conventional MEA (i.e., based on Pt/C). As shown in Figure 6i, in the high‐voltage region, where the catalytic effect is dominant, RuPt_SA_/C presents a comparable performance to Pt/C due to the fact that the presence of sufficient OH_ad_ on the Ru surface accelerates the HOR. Although RuPt_SA_/C exhibited a slightly lower maximum power density (i.e., (0.39 W cm^−2^) compared to that of Pt/C (i.e., 0.42 W cm^−2^), its current density at 0.6 V was 0.57 A cm^−2^, which is identical to that of Pt/C (Figure 6j). Moreover, the low‐voltage region, where RuPt_SA_/C begins to degrade, is typically dominated by mass transport, and the decrease in performance in this region can be attributed to inhibited mass transport. Owing to the hydrophilic properties of Ru, a large amount of water became adsorbed onto the catalyst surface, thereby hindering contact with hydrogen. Thus, although a slight performance degradation was observed due to the mass transport resistance in the low‐voltage region, the obtained results suggest that RuPt_SA_/C containing an ultralow Pt loading and exhibiting a high HOR activity and an excellent impurity tolerance, shows considerable potential for use as a fuel cell anode catalyst in both AEMFCs and PEMFCs.

## 3 Conclusion

Current catalysts for the anodes of fuel cells tend to be highly dependent on the use of Pt, which is prone to poisoning by impurities. Moreover, the performances of non‐Pt‐based catalysts in single‐cell applications tend to be poor. In this work, a strategy was introduced to achieve an excellent hydrogen oxidation activity and a high poisoning resistance by developing low‐cost catalysts wherein single Pt atoms were introduced onto the surfaces of Ru nanoparticles. By favorably reconstructing the active sites on the Ru surfaces using ultralow quantities of Pt, the synergy between the intrinsic characteristics of Pt (i.e., moderate hydrogen and CO binding energies) and Ru (i.e., oxophilicity) was fully exploited. The resulting RuPt_SA_/C catalyst not only exhibited an excellent mass activity in the hydrogen oxidation reaction (HOR), but it also demonstrated a significantly higher CO resistance. Notably, the mass activity of this catalyst was 25.4‐ and 18‐fold higher than those achieved by Pt/C in proton exchange membrane fuel cells (PEMFCs) and anion exchange membrane fuel cells (AEMFCs), respectively. These properties of the RuPt_SA_/C catalyst were confirmed by density functional theory calculations of the hydrogen and CO adsorption energies in the context of the HOR, and they were verified by practical PEMFC and AEMFC device evaluations. Overall, the results presented herein suggest that the introduction of single atoms via galvanic substitution is a low‐cost strategy for maximizing the benefits of both metals, and it also provides novel insights into the design of fuel cell anode catalysts.

## 4 Experimental Section

The materials and methods are described in Section S1.

## Conflicts of Interest

The authors declare no conflicts of interest.

## Supporting information




**Supporting File**: smll75024‐sup‐0001‐SuppMat.docx.

## Data Availability

The data that support the findings of this study are available from the corresponding author upon reasonable request.
