# Supplementary material for: Reconfiguration of Localized Ruthenium Surface via Incorporating Single Platinum Atoms for Favorable Hydrogen Oxidation Catalysis
Source: Small. 2026 Aug 4;22(47):e75024. doi: 10.1002/smll.75024 (PMC13495821; doi:10.1002/smll.75024)
Supplement: Supplementary file 1 [file SMLL-22-e75024-s001.docx]

Supporting Information

**Reconfiguration of Localized Ruthenium Surface via Incorporating Single Platinum Atoms for Favorable Hydrogen Oxidation Catalysis**

*Daeil Choi^1,2^* ^†^*, Dong Wook Lee^1,3^* ^‡^*, Hochang Song^4^, Seung-hoon Kim^1^, Dohoon Kim^5^,* *Jongkyung Ryu^6^, Jue-Hyuk Jang^1^, Sungyong Cho^3^, Sungchul Lee^3^, Segeun Jang^5^, Hyung Chul Ham^4^, and Sung Jong Yoo^1,7^*

**Table of Contents**

1. Section S1

2. Figure S1-S18

3. Table S1-S5

Section S1. Materials and methods

*Materials:* Platinum(IV) chloride (PtCl_4_), ruthenium(III) chloride hydrate (RuCl_3_ꞏxH_2_O), sodium borohydride (NaBH_4_), oleyamine (technical grade, 70%), and Nafion perfluorinated resin solution (5 wt.%) were purchased from Sigma-Aldrich. Ethanol and isopropyl alcohol (IPA) were obtained from Merck (Darmstadt, Germany). Commercial Pt/C (20 wt.% Pt on carbon, Johnson Matthey) was used as the reference catalyst for physical characterization, RDE electrochemical measurements, and anode-side single-cell evaluations. Commercial Pt/C (46.5 wt.% Pt on carbon, TANAKA) was used as the cathode catalyst in the single-cell tests. All materials were used as received without further purification.

*Preparation of the Ru/C and RuPt_SA_/C catalysts:* The Ru/C catalyst was prepared via a common wet chemical synthesis method. More specifically, carbon black (100 mg) and oleylamine (2 mL) were evenly dispersed in ethanol (400 mL) for 2 h under ultrasonication conditions. Subsequently, RuCl_3_ꞏxH_2_O (0.247 mmol) and NaBH_4_ (281 mg) were added, and the resulting mixture was held at 25 °C for 10 h under vigorous stirring. After this time, the obtained mixture was filtered and washed with ethanol. Two heat-treatment steps were then conducted to ensure effective oleylamine removal. Firstly, the as-prepared Ru/C was annealed under air at 250 °C for 2 h, and subsequently, continuous heat treatment was carried out in a H_2_ atmosphere to reduce the surfaces of the Ru nanoparticles. The desired RuPt_SA_/C catalyst was then obtained using the galvanic displacement method. For this purpose, the prepared Ru/C catalyst (50 mg) was dispersed in deionized (DI) water (500 mL) for 1 h under ultrasonication conditions. Subsequently, PtCl_4_ (0.005 mmol) was added to the above solution under stirring, and the resulting mixture was held at 25 °C for 10 h under vigorous stirring. Finally, the obtained mixture was washed several times with DI water to any remove impurities, and subsequently dried.

*Physical characterization:* Quantitative analyses of the metal components (Pt and Ru) present in the electrocatalysts were performed using inductively coupled plasma–mass spectroscopy (ICP-MS; ELAN 6100, PerkinElmer, Inc.). High-resolution transmission electron microscopy (HR-TEM; Talos F200X and Titan TM, FEI) was used to characterize the catalyst morphologies, while energy dispersive X-ray spectroscopy (EDX) in the STEM mode was used to analyze the line scan profiles at an acceleration voltage of 200 keV. The samples for these studies were prepared by dispersing adequate amounts of the catalyst in ethanol using an ultrasonic bath, followed by dropping a small amount of this dispersed mixture onto a carbon-hollow Cu grid and drying in an oven. XRD analyses of the catalysts were conducted using a Rigaku D/Max 2500 diffractometer operated with Cu Kα radiation (λ = 1.54056 Å) at 60 kV and 300 mA. The diffraction patterns of the samples were obtained in a 2θ range of 20–80° with a scan rate of 2° min^−1^. The EXAFS and XANES analyses were performed at the 8C nanoprobe XAFS beamline (BL8C) of the Pohang Light Source (PLS-II) in a 3.0 GeV storage ring with a ring current of 250 mA. The X-ray beam was monochromated by a Si(111) double crystal where the beam intensity was reduced by 30% to eliminate the higher-order harmonics. The X-ray beam was then delivered to a secondary source aperture, where the beam size was adjusted to 0.5 mm (v) × 1 mm (h). XPS (Thermo Sigma Probe, Thermo Fisher Scientific, Waltham, Massachusetts, USA) was conducted using an Al Kα X-ray beam source. All spectra were deconvoluted using XPSPEAK 4.1 software, and calibrated with the C 1s peak at 284.6 eV.

*Electrochemical measurements:* The electrochemical properties were measured using a three-electrode workstation system (Autolab, Ecochemie), and all potential values were presented versus the RHE in the half-cell data. A Pt wire and a saturated calomel electrode (SCE) were used as the counter and reference electrodes, respectively. A glassy carbon rotating disk electrode (RDE) with a diameter of 5 mm (geometric area = 0.196 cm^−2^) was used as the working electrode. To prepare the catalyst ink, a portion of each catalyst (10 mg) and an aliquot of a 5 wt.% Nafion solution (100 μL) were added to IPA (1 mL) and subjected to sonication for 15 min. After this time, a portion (5 μL) of the catalyst ink was coated onto the GC electrode and dried under ambient conditions. The loading densities of Pt were 59.32 μg_Pt_ cm^−2^ (Pt/C) and 2.27 μg_Pt_ cm^−2^ (RuPt_SA_/C). The HOR activities of the various catalysts were evaluated in H_2_ and in a 10 ppm CO/H_2_-saturated 0.1 M HClO_4_ environment at 4 ℃ and 1600 rpm. The potential range was set from −0.05 to 1.0 V. Cyclic voltammetry (CV) was carried out for all catalysts in Ar-saturated 0.1 M HClO_4_ at a scan rate of 20 mV s^−1^. For the CO stripping, CO molecules were adsorbed onto the electrode surfaces at 0.05 V by injecting 99.9% CO gas for 15 min, followed by the removal of CO gas by dissolving in the electrolyte under Ar purging for 30 min. CO stripping was performed in a 0.1 M HClO_4_ solution in the potential range of 0.05–1.05 V with a 20 mV s^−1^ scan rate. For analysis of the CO bulk oxidation process, the measurement conditions employed for CO stripping was used, with the exception of a rotational speed of 1600 rpm and the introduction of a continuous CO gas feed. Time-transient CO bulk oxidation tests were also performed at 0.5, 0.6, 0.7, and 0.8 V with continuous CO injection. The HOR polarization curves were measured in alkaline electrolytes (0.1 M KOH and tetramethylammonium hydroxide (TMAOH)) at 25 ℃. Chronoamperometry was conducted at 0.1 V vs. RHE for 120 min to adsorb the organic cations onto the catalyst surface.

*Computational methods:* Spin-polarized DFT calculations were performed using the Perdew–Burke–Ernzerhof (PBE) method, as implemented in the Vienna ab initio simulation package (VASP). ^[1–3]^ The projector augmented wave (PAW) method using a plane-wave basis set was implemented to describe the interaction between the core and valence electrons. ^[4]^ The Perdew–Burke–Ernzerhof (PBE) exchange–correlation functional within a generalized gradient approximation (GGA) was employed. ^[5]^ For the plane wave expansion of the electronic eigenfunctions, an energy cutoff of 400 eV was used. Initially, an optimized unit cell was obtained for the hcp-structured (P63/mmc) Ru bulk with lattice parameters of a = b = 2.71 Å, and c = 4.28 Å, which were similar to the experimental results of a = b = 2.71 Å, and c = 4.29 Å.32 Using this optimized bulk unit cell, the five-layered (2 × 2) Ru(0001) slab model was prepared and isolated from its periodic images in the vertical direction by the vacuum distance corresponding to seven atomic layers. ^[6]^ While the bottom two layers were fixed at the corresponding bulk positions, the upper three layers were fully relaxed using the conjugate-gradient method until the residual forces on all atoms became <5 × 10^−2^ eV Å^−1^. To model the RuPt_SA_/C surface, one of the top Ru atoms was replaced by Pt. For the Brillouin zone integration, (16 × 16 × 16) and (5 × 5 × 1) Monkhorst-Pack meshes of k-points were used to determine the equilibrium geometries and total energies of the Ru bulk and slab models. In addition, the k-points were increased to (10 × 10 × 1) for the surface electronic structure calculations. The average d-band energies (d-band center, μ) for the Ru(0001) and RuPtSA/C surfaces were calculated as follows:

$$\mu=\frac{\int_{-\infty}^{\infty} \left( \varepsilon\times\rho\right)d\varepsilon}{\int_{-\infty}^{\infty} \rho d\varepsilon}$$

where ρ and ε represent the electron density (ρ) at a certain energy level (ε). The average atomic charge density relative to the bulk atom (σ) was calculated using the decomposition scheme proposed by Bader.

The Gibbs free energy differences (ΔG) under standard reaction conditions (T = 298.15 K, P = 1 bar) were calculated as follows to predict the adsorption energy and the HOR onset potential:
 ΔG = ΔE − TΔS + ΔZPE

where ΔE, ΔS, and ΔZPE represent the total energy change directly obtained from the DFT calculations, the entropy change, and the change in the zero-point energy, respectively. The entropy of the gaseous molecules under reaction conditions was taken from the NIST Chemistry WebBook, ^[7]^ and the entropy and zero-point energy of the adsorbed molecules on the surface were computed from the vibrational frequencies, in which the adsorption vibrational mode was calculated explicitly with all atoms fixed except for the adsorbed molecules. ^[8]^ The adsorption energies of CO, H, and TMA^+^ on the RuPt_SA_/C, Ru(0001), and Pt(111) surfaces were calculated using the following equation:

ΔE_Ads_(X) = E_X*_ − [E_slab_ + E_X_]

where E_X*_, E_slab_, and E_X_ represent the total energies of the slab containing adsorbed X species, the clean slab, and the isolated X molecule, respectively. ΔE_Ads_(X) was calculated with OH groups on the surface considering alkaline conditions.

*Single-cell tests:* For the PEMFCs, a MEA was prepared using an ultrasonication-assisted electrospray device (ESR200R2, NanoNC). ^[9]^ The geometrically active area of the catalyst coating layer was 5 cm^2^ and commercial Pt/C (46.5 wt.%, Tanaka) was deposited with a loading density of 0.4 mg_Pt_ cm^−2^ at the cathode for all MEAs. For the anode, commercial Pt/C (20 wt.%, Alfa-Aesar) and RuPt_SA_/C were applied with loading densities of 0.2 and 0.019 mg_Pt_ cm^−2^, respectively. The polarization curve measurements were carried out for the combined cell at 60 °C by maintaining a humidified gas flow (100% relative humidity) of H_2_ (and 10 ppm CO/H_2_) and air (200 cc min^−1^ for the anode, 600 cc min^−1^ for the cathode). For the AEMFCs, RuPt_SA_/C and Pt/C (Pt 20 wt.%, Premetek, USA) were used separately as anode catalysts. Each catalyst was mixed with DI water, IPA, and the FAA-3 ionomer (10 wt.%, Fumatech, Germany). After the preparation of a well-dispersed slurry under ultrasonication conditions, the slurry was sprayed onto an FAA-3 membrane (20 μm, Fumatech, Germany) using an air brush to give a deposition density of 0.4 mg_PGM_ cm^−2^ at the anode. A mixture of Pt/C (46.9 wt.% Pt, Tanaka, Japan), DI water, IPA, and the FAA-3 ionomer was used to prepare the cathode catalyst slurry for the reference MEA. Using an air brush, the ultrasonically dispersed cathode catalyst slurry was sprayed to give a deposition density of 0.5 mg_PGM_ cm^−2^. All electrodes had an active area 5 cm^2^. To ensure the incorporation of impurity anions, the fabricated MEAs were immersed in 1.0 M KOH twice for 30 min and then for 150 min. Prior to assembly of the cell components, the KOH-pretreated MEAs were rinsed with DI water. Each MEA was assembled between gas-diffusion layers (Sigracet 39BB, SGL Carbon, Germany), gaskets, and graphite bipolar plates with a serpentine channel. To determine the electrochemical characteristics of each system, a fuel-cell test station (CNL Energy, KOREA) and a potentiostat (HCP 803, BioLogic, France) were used. During the measurements, humidified H_2_ (0.8 slpm, RH 80) and O_2_ (1.0 slpm, RH 92) were supplied to the anode and cathode at a cell temperature of 60 °C. For the *I-V* curves, the voltage sweep method was employed with a scan rate of 4 mV s^−1^ from the open circuit voltage to 0.30 V. At a current density of 200 mA cm^−2^, the EIS spectra were acquired at 0.85 and 0.45 V following the *I-V* curve measurements.

**Figure S1**. TEM images of Ru/C (a) before and (b) after heat treatment with different resolution. (Air-250 ℃ for 1h; H_2_-300 ℃ for 1h)

**Table S1.** ICP-MS analysis for the quantification of Ru/C and RuPt_SA_/C.

**Figure S2**. Particle diameter distribution of RuPt_SA_.

**Figure S3**. STEM image of RuPt_SA_ (yellow circles indicate the single Pt atom sites).

**Figure S4**. Areal ratios of the various Pt 4f and Ru 3d states in the XPS spectra recorded for the Pt/C and RuPt_SA_/C species.

**Table S2.** Comparison of the binding energy and atomic ratio of each state (Pt^0^, Pt^2+^ and Pt^4+^)

**Table S3.** Comparison of the binding energy and atomic ratio of each state (Ru metal, RuO_2_ and RuO_x_H_y_).

**Figure S5**. (a) Micro-polarization region and (b) the change of current density at 5 mV and limiting current density in HOR polarization curves of Pt/C and RuPt_SA_/C under pure H_2_ and CO 10 ppm/H_2_-saturated 0.1 M HClO_4_

**Figure S6**. The change of current density at 50 mV in HOR polarization curves of Pt/C and RuPt_SA_/C under pure H_2_ and CO 10 ppm/H_2_-saturated 0.1 M HClO_4_.

**Figure S7.** Background-corrected CO bulk oxidation results measured in CO-saturated electrolyte at a scan rate of 20 mV s^-1^ at 1600 rpm.

**Figure S8.** The time-transient bulk CO oxidation of Pt/C and RuPt_SA_/C which is measured at (a) 0.5, (b) 0.6, (c) 0.7, and (d) 0.8 V in CO-saturated 0.1 M HClO_4_.

**Figure S9.** (a) Chronoamperometry at 0.1 vs. RHE and (b) current density decrease rate of commercial Pt/C and RuPt_SA_/C. All measurement was conducted in10 ppm CO/H_2_ saturated 0.1 M HClO_4_ with rotational speed of 1600 rpm for 10000s.

**Table S4.** The comparison of onset and peak potential of Pt/C, Ru/C and RuPt_SA_/C from CO stripping and CO bulk oxidation measurement.

**Figure S10**. Optimized CO-adsorbed structures of Ru(0001), Pt(111), and RuPt(0001) according to OH coverage.

**Table S5.** Hydrogen and CO adsorption energy of Ru(0001), Pt(111), and RuPt(0001) slab models in clean and OH-adsorbed surface.

**Figure S11**. Tafel plots and slopes of Pt/C, Ru/C, and RuPt_SA_/C of HOR polarization curves in 0.1 M KOH.

**Figure S12**. Micro-polarization region of HOR polarization curves of Pt/C, Ru/C, and RuPt_SA_/C.

**Figure S13**. HOR polarization curves of Pt_SA_/C and RuPt_SA_/C

**Figure S14.** The morphology of carbon-supported Pt single atom (Pt_SA_/C)

**Figure S15.** EXAFS spectrum of carbon supported Pt single atom (Pt_SA_/C), comparing to that of Pt foil.

**Figure S16.** XPS spectrum of carbon supported Pt single atom (Pt_SA_/C).

**Figure S17**. Micro-polarization region and in HOR polarization curves of Pt/C and RuPt_SA_/C under pure H_2_ and CO 10 ppm/H_2_-saturated 0.1 M KOH.

**Figure S18**. The change of current density at 50 mV in HOR polarization curves of Pt/C and RuPt_SA_/C under pure H_2_ and CO 10 ppm/H_2_-saturated 0.1 M KOH.

**References**

[1]	P. Hohenberg, W. Kohn, *Phys. Rev.* **1964**, *136*, B864.

[2]	W. Kohn, L. J. Sham, *Phys. Rev.* **1965**, *140*, A1133.

[3]	G. Kresse, J. Furthmüller, *Phys. Rev. B* **1996**, *54*, 11169.

[4]	P. E. Blöchl, *Phys. Rev. B* **1994**, *50*, 17953.

[5]	J. P. Perdew, K. Burke, M. Ernzerhof, *Phys. Rev. Lett.* **1997**, *78*, 1396.

[6]	J. M. Kim, Y. J. Lee, S. Kim, K.-H. Chae, K. R. Yoon, K. A. Lee, A. Byeon, Y. S. Kang, H.-Y. Park, M. K. Cho, *Nano Energy* **2019**, *65*, 104008.

[7]	NIST Standard Reference Database Number 69, https://webbook.nist.gov/chemistry/

[8]	E. Skúlason, V. Tripkovic, M. E. Björketun, S. Gudmundsdóttir, G. Karlberg, J. Rossmeisl, T. Bligaard, H. Jónsson, J. K. Nørskov, *J. Phys. Chem. C* **2010**, *114*, 18182.

[9]	J. Lee, J. Seo, K. Han, H. Kim, *J. Power Sources* **2006**, *163*, 349.
